# Invading and Expanding: Range Dynamics and Ecological Consequences of the Greater White-Toothed Shrew (*Crocidura russula*) Invasion in Ireland

**DOI:** 10.1371/journal.pone.0100403

**Published:** 2014-06-23

**Authors:** Allan D. McDevitt, W. Ian Montgomery, David G. Tosh, John Lusby, Neil Reid, Thomas A. White, C. Damien McDevitt, John O'Halloran, Jeremy B. Searle, Jon M. Yearsley

**Affiliations:** 1 School of Biology and Environmental Science, University College Dublin, Dublin, Ireland; 2 School of Biological Sciences, Queen's University Belfast, Belfast, Northern Ireland; 3 Quercus, School of Biological Sciences, Queen's University Belfast, Belfast, Northern Ireland; 4 Birdwatch Ireland, Kilcoole, Co. Wicklow, Ireland; 5 Department of Ecology and Evolutionary Biology, Cornell University, Ithaca, New York, United States of America; 6 Department of Biology, University of Oulu, Oulu, Finland; 7 Deele College, Raphoe, Co. Donegal, Ireland; 8 School of Biological, Earth and Environmental Sciences, University College Cork, Cork, Ireland; University of Western Ontario, Canada

## Abstract

Establishing how invasive species impact upon pre-existing species is a fundamental question in ecology and conservation biology. The greater white-toothed shrew (*Crocidura russula*) is an invasive species in Ireland that was first recorded in 2007 and which, according to initial data, may be limiting the abundance/distribution of the pygmy shrew (*Sorex minutus*), previously Ireland's only shrew species. Because of these concerns, we undertook an intensive live-trapping survey (and used other data from live-trapping, sightings and bird of prey pellets/nest inspections collected between 2006 and 2013) to model the distribution and expansion of *C. russula* in Ireland and its impacts on Ireland's small mammal community. The main distribution range of *C. russula* was found to be approximately 7,600 km^2^ in 2013, with established outlier populations suggesting that the species is dispersing with human assistance within the island. The species is expanding rapidly for a small mammal, with a radial expansion rate of 5.5 km/yr overall (2008–2013), and independent estimates from live-trapping in 2012–2013 showing rates of 2.4–14.1 km/yr, 0.5–7.1 km/yr and 0–5.6 km/yr depending on the landscape features present. *S. minutus* is negatively associated with *C. russula*. *S. minutus* is completely absent at sites where *C. russula* is established and is only present at sites at the edge of and beyond the invasion range of *C. russula*. The speed of this invasion and the homogenous nature of the Irish landscape may mean that *S. minutus* has not had sufficient time to adapt to the sudden appearance of *C. russula*. This may mean the continued decline/disappearance of *S. minutus* as *C. russula* spreads throughout the island.

## Introduction

Invasive species can have dramatic and rapid impacts on faunal and floral communities, either through direct competition or through indirect effects such as trophic cascades [1]–[3]. Establishing how invaders impact upon pre-existing species (e.g. potentially driving some to extinction [4]) is a fundamental question in ecology and conservation biology [5], and is vital for the management of biological invasions [6]. The potential impact of competition during invasions can be underestimated if we compare it to competition within communities that are static, since competition is expected to decrease over time as species co-evolve [4], [7]. The strength of any competitive effects between invasive and pre-existing species may depend on the community composition of the invaded environment (as well as the environment itself [4], [8]), the speed of the invasion, and trade-offs between dispersal, reproduction and competitive ability of the invasive species as it expands its range [9]–[11].

Interspecific competition between species will be influenced by species traits and features of the local environment [4], [8], [12]. Shrews are small, ground-dwelling, insectivorous mammals that have been well-studied in terms of competitive interactions. For them, it has been proposed that differences in body size between species can drive the segregation of species into habitats of differing productivity, with larger species being most abundant in more productive habitats [12], [13]. Competition between shrew species has primarily been studied in regions where species naturally overlap [12]–[16]. Competition directly following the establishment of a new species has not been studied, perhaps because there have been few documented cases of shrews being considered as an invasive species [17]. Such a species invasion has occurred on the island of Ireland with the recent arrival of the greater white-toothed shrew (*Crocidura russula*).

*C. russula* was discovered in Ireland from pellets of barn owls (*Tyto alba*) and kestrels (*Falco tinnunculus*) collected in 2007, with later confirmation by live-trapping in 2008 [18]. *C. russula* is distributed in northern Africa and western Europe, and was previously absent from the British Isles [19], [20]. The pygmy shrew (*Sorex minutus*) is present throughout the British Isles (and distributed widely in Europe) and was notably the only shrew species present on Ireland until the arrival of *C. russula*. The human-mediated introduction of *S. minutus* to Ireland dates back to the Neolithic period [21], [22]. *C. russula* is approximately three times the size of *S. minutus*, is gregarious (as opposed to the highly territorial and relatively solitary lifestyle of *S. minutus*), and has smaller home range sizes than *S. minutus* [16], [20], [23]. At first, it was proposed that the introduction of *C. russula* could prove beneficial to the Irish ecosystem as a prey item for birds of prey [18]. However, trapping conducted on sites within the Irish range of *C. russula* in winter 2010/2011, found that no *S. minutus* were present at these sites [24]. These two species are sympatric in western Europe [19], with *S. minutus* generally being uncommon where they occur together (representing 0.7–2.9% of small mammal catches in various habitats in France for example [25]–[27]) but may be locally more numerous, in particular on the island of Belle Île off the coast of northern France ([27] Fig. S1). This suggests that the interaction between the two species in Ireland may be different from mainland Europe and the islands where both species have been long-established. This highlights the difficulty in predicting the outcome of species' interactions arising from species' introductions [4].

An accurate assessment of its current distribution and the rate at which *C. russula* is spreading are needed due the potential negative impact of the species. The range expansion of another invasive small mammal in Ireland, the bank vole (*Myodes glareolus*), which arrived in the early 1900s, has been the subject of a number of studies [28]–[31] but similar analysis is lacking for *C. russula*. Previously published data estimated the range of *C. russula* to be approximately 2,300 km^2^ in the winter of 2010/2011 [24] but this was a minimum estimate and not the primary focus of the study in question. The length of time that the invasive shrew has been present in Ireland and the rate at which it is expanding its range is pivotal for our understanding of how community dynamics will change in Ireland's small mammal communities.

If *C. russula* is having a negative impact on *S. minutus* in Ireland, data from localities where the two species are still occurring together in Ireland are crucial for understanding any replacement processes. Such data have been lacking previously [24]. Similarly, distributions and abundances of all species already present in the invaded community may be important in predicting the individual and cumulative impact of the invasive species. Montgomery et al. [24] reported that there was a combined impact of *C. russula* and *M. glareolus* upon *S. minutus* and another small mammal, the woodmouse (*Apodemus sylvaticus*) in Ireland. Like *S. minutus*, *A. sylvaticus* is probably another early human-mediated introduction to Ireland, first appearing in the Mesolithic [32]. This combined impact of *C. russula* and *M. glareolus* conformed to the concept of ‘invasional meltdown’ [33], where the presence of one invading species facilitates another and compounds the negative impacts on pre-existing species, communities and ecosystems. Therefore, in order to assess the impact of the recently invading species (*C. russula* and *M. glareolus*) on the pre-existing species (*S. minutus* and *A. sylvaticus*), it is necessary to tease out the influence of each species (as well as the effects of their interactions) on each other and the influence of the surrounding environment [13], [24]. The species considered here overlap in their diet [24], and there is considerable overlap in habitats throughout their European ranges although there is variation from place to place, e.g. *S. minutus* is absent from forests in a multi-shrew community in France [25] but is abundant in forests in Ireland [27], [34]. Therefore, the arrival of a new competitor could potentially alter the habitat preferences of the existing species [4].

The objective of the research we report here is to assess the immediate impact of *C. russula*, and to predict the rate at which these impacts will spread across the island of Ireland. More precisely, we have two aims. The first aim is to establish the current distribution of *C. russula* in Ireland and to estimate the rate of range expansion. The second aim is to assess the impact of *C. russula* on the small mammal community in Ireland by establishing single species and interaction associations, as well as the potential influence of habitat on each species.

## Materials and Methods

### Ethics Statement

All species were live-trapped with approval from the National Parks and Wildlife Service (NPWS) in Ireland and Animal Research Ethics Committee in University College Dublin (AREC-13-24). *C. russula* were euthanized by cervical dislocation in accordance with instructions given by the NPWS of a species not ‘ordinarily resident in the State’. *S. minutus* is a protected species in the Republic of Ireland and was trapped according to a license issued by the NPWS (License no. C157/2011).

### Distribution, abundance and rate of expansion of *C. russula*

**Figure 1:**
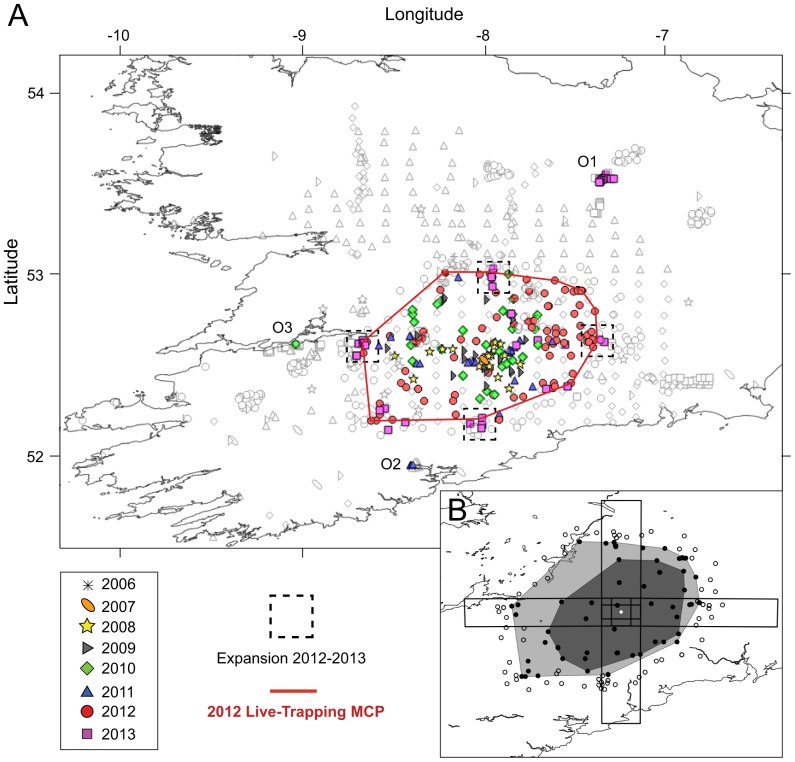
Distribution of *Crocidura russula* in Ireland. Positive (filled shapes) and negative (empty shapes) *Crocidura russula* records in Ireland from 2006 to 2013 (A). Localities O1–3 are outlier records (see main text). Dashed squares represent sites that were used for the estimation of range expansion between 2012 and 2013 (A). The 100% minimum convex polygon (MCP) from the 2012 summer live-trapping survey is shown in red (A). Inset map (B) shows this same MCP, highlighting the three ‘Zones’ used for the analyses of species interactions and habitat associations, and the four transects used to measure changes in species abundance and expansion rate. Black circles are *C. russula* positive and white circles are *C. russula* negative sites in 2012. Zone 1 (dark grey) is the MCP of *C. russula* sites which contained no *Sorex minutus* observations, Zone 2 (light grey) is the MCP of all *C. russula* observations, Zone 3 (the rest of the region) is outside the observed range of *C. russula*. The white diamond represents the centroid of Zone 1.

All available sighting and live-trap data on *C. russula* (with associated dates) were collated for 2006–2013 (Figs 1A and S2; Table S1) based on records from the National Biodiversity Data Centre in Ireland (www.biodiversity.ie) and small mammal trapping studies in the region [18], [24], [30], [31]. In addition, data on prey identification from barn owl and kestrel pellets and identification of prey remains from nest inspections were also included [35]–[37]. Both predators are known to feed on the range of small mammal species present in Ireland (including *C. russula* [18]). They are central-place foragers which after a period of post-fledging dispersal are largely sedentary within a relatively small home range and show a high level of fidelity to specific nest and roost sites within that range [38]–[40]. Available data on *C. russula* from the scats of pine martens (*Martes martes*) were not included because the exact sampling dates were not known [41]. We used all available data to provide an initial estimate of the expansion rate for *C. russula* (km/yr). We partitioned the data by date, making sure that data from one survey were not split between subsets (date bins were 2008, 2009, Jan-Sept 2010, Oct 2010–Feb 2011, Mar 2011–Mar 2012, Apr 2012–Dec 2012, 2013). For each subset we calculated the area, *A*, of the minimum convex polygon (MCP) for *C. russula* and fitted a linear regression of the radial range of the species distribution (km) against the median date (yr) of the data points in each subset. Our resulting estimate of the radial expansion rate is the slope of this regression line.

A more focused study established the distribution of *C. russula* in the summer of 2012 and its expansion after one year (2013; Table S2). Trapping started on or adjacent to the boundary of the range defined by Montgomery et al. [24] and was conducted during May 31–September 2, 2012, and June 19–24, 2013. Trap lines were set in each 10 km square (standard Irish grid hectads) in hedgerows adjacent to agricultural land (the most prevalent small mammal habitat available in Ireland [24]). Trap lines consisted of 26–112 (mean = 39) Trip-Traps (Proctor Bros. Ltd.), spaced approximately 5 m apart and baited with blowfly larvae and oat flakes. Trap lines were left for 18–24 h to cover a cycle of diurnal and nocturnal activity. Trapping continued outwards from the range defined by Montgomery et al. [24] until *C. russula* was completely absent at two sites roughly in parallel. The numbers of all small mammals caught were recorded. Once the distribution of *C. russula* was established, further trapping was conducted within the range of *C. russula* and at the edge of that range where the species occurred together with *S. minutus*. A total of 123 sites were surveyed in 2012. In 2013, trapping was conducted at a further 20 sites. Trapping in 2013 was clustered around four areas on the 2012 range boundary (Fig. 1A) so that we could estimate the rate of range expansion during 2012–2013.

In order to investigate changes in the number of *C. russula* caught per trap (hereafter referred to as abundance) at different points in its range and the rate at which it has been expanding, clines were fitted along four transects running east, west, north and south from the centroid of Zone 1 (Zone 1 being the core range of *C. russula* within which no *S. minutus* were caught, see below and Fig. 1B). We also fitted clines for the other species, as a measure of the impact of the *C. russula* expansion on them. We *a priori* fixed the functional form of the cline because of the small number of data points within any one transect (n<16). For *C. russula*, *A. sylvaticus* and *M. glareolus* we fitted sigmoidal clines of the form
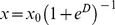
(1a)but for *S. minutus* the cline was so abrupt that we fitted a step function of the form
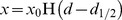
(1b)where
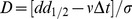
(2)and H(*x*) is the Heaviside step function, *x_0_* is the asymptotic number of individuals per trap far from the cline, *d* is the distance (km) of a point from the centroid of Zone 1, *d_1/2_* is the value of *d* at which *x* = *x*_0_/2 in the year 2012, *v* is the velocity at which the cline is moving (km/yr), *σ* is the width of the cline (km) and Δ*t* is the time since 2012 (yr). The functions (1a) and (1b) were fitted using non-linear least squares to the 2012 and 2013 data (Method 1 in Table 1 for estimating velocity, *v*). For *C. russula* we estimated the time taken to disperse from *d* = 0 to the point in 2012 when *C. russula* abundance was 0.1 *x_0_*. This was done using the point estimates and standard errors for *d_1/2_*, *σ* and *v*, to generate 1000 Monte Carlo realizations for this invasion time from which the distribution of invasion times was estimated.

**Table 1:** Expansion rates and time since expansion.

Transect	Velocity (SE) (Method 1)	Median Time (Q0.25–Q0.75)	Year (Q0.75–Q0.25)	Velocity (SE) (Method 2)	Velocity (Method 3)
North	4.1 (1.8)	10.1 (7.9–14.1)	2002 (1998–2004)	4.3 (1.6)	3.1
South	14.1 (6.8)	3.7 (1.6–7.2)	2008 (2005–2010)	7.1 (3.3)	5.6
East	5.7 (1.3)	5.7 (4.8–6.8)	2006 (2005–2007)	3.0 (2.1)	1.5
West	2.4 (2.2)	16.8 (11.3–30.7)	1995 (1981–2001)	0.5 (1.7)	0

Estimates of velocity (km/yr), median time since expansion (with 0.25 and 0.75 quantiles) and the year when the *Crocidura russula* population expanded for each transect for Method 1 (Fig. 1B). Methods 1, 2 and 3 are described in the main text.

The estimates of velocity from the clines (equations 1 and 2) assume a shape for the cline and that this shape does not change between years. We used generalized additive models (Method 2 in Table 1) to test if our estimates of velocity were robust to the shape of the cline at the invasion front. Using data points that were at least 25 km from the centroid of Zone 1 (in order to focus upon the invasion front) we fitted thin plate regression splines to the shape of the invasion front. The shape of the fitted cline is therefore driven by the data. The velocity of the cline was then estimated from the shift in the cline's position from 2012 to 2013, assuming that the shape of the cline does not change (see File S1). Finally, we used a third method (Method 3 in Table 1) to give point estimates of the rate of spread of *C. russula* that did not rely upon fitting a cline to the data. This method found the *C. russula* observations along each transect that were furthest from the centroid in 2012 and 2013. The difference in distance between 2012 and 2013 was used as a point estimate for the rate of expansion.

### Habitat associations and species interactions

To investigate the effect of land-use and species-interactions on small mammal abundances, we defined three spatial regions based upon the distributions of *C. russula* and *S. minutus* from 2012 (Fig. 1B). The first region (called Zone 1) contained all sites where *C. russula* were caught and whose MCP contained no *S. minutus* captures (30 sites). The second region (called Zone 2) contained all sites that were not in Zone 1 but were within the MCP of *C. russula* captures (40 sites). Finally, Zone 3 contained all sites not in Zone 1 or Zone 2 (53 sites, by definition there were no *C. russula* captures in Zone 3). The land cover for our sampling region was extracted from the Corine Land Cover 2006 seamless vector data set (Version 16 (04/2012)) http://www.eea.europa.eu/data-and-maps/data/clc-2006-vector-data-version-2. We defined four aggregate land cover classes as broad proxies for variation in small mammal habitat and which were the dominant land covers around our sampling locations: forest (broad-leaved, coniferous and mixed woodland; Corine codes 311, 312 and 313), arable (Corine codes 211, 242 and 243), pasture (Corine code 231), natural grassland (Corine codes 321, 322, 324). We then calculated the proportion of each land-cover class in two circular rings (buffers) around each sampling location (within a 500 m radius and from 500 m to 2000 m in radius). These spatial scales were chosen to reflect the home range sizes and average dispersal distances in *C. russula* (within 500 m [20], [23], [42], up to the maximum dispersal distance recorded for the species (1.3 km [42]). Therefore, shrews within a single generation could sample a range of habitats within these spatial scales. All spatial data were manipulated using the sp, rgdal, and rgeos packages in R. Correlations between land covers where avoided by using only forest, grass and arable land cover classes (because pasture represented between 72% to 76% of land cover at each spatial scale and was negatively correlated with the sum of forest, grass and arable land covers).

We built spatial models for the abundances of each small mammal species at each sampling location within the range of a species (Table S2; Fig. 1B). The models for *C. russula* and *S. minutus* are therefore based upon different datasets with different spatial extents (Zones 1 and 2 for *C. russula* and Zones 2 and 3 for *S. minutus*). All models were fitted by maximum likelihood using generalised least squares (using the nlme package in R) with an exponential spatial error structure. The response variable was square-root transformed to compensate for over-dispersion. We defined a maximal model for each species (File S1; Table S4) and performed model averaging [43] across all combinations of explanatory variables (Table S3; all continuous explanatory variables were scaled to have a mean of zero and unit variance). Models always contained three control variables: the number of traps (NumTrap), the phase of the moon (Lunar) and a categorical factor for whether or not it had rained during the trapping session (Rain) as these variables have been previously demonstrated to influence the trapping success of small mammals [24], [44], [45]. The model with the lowest AICc was selected as the ‘best approximating’ model and all models with a ΔAICc<2 from the ‘best approximating’ model were selected for model averaging (Tables S5–S12). The Akaike weight of each model was calculated, and normalised so that the weight of all selected models summed to one. The fitted coefficients for the explanatory variables were then averaged across the selected models. The uncertainty in an averaged coefficient was estimated by the unconditional variance estimator. The overall relative importance of each explanatory variable was quantified by summing the Akaike weights (Σ*ω_i_*) of selected models in which an explanatory variable occurred (*w_+_*).

## Results

### Distribution and expansion

**Figure 2:**
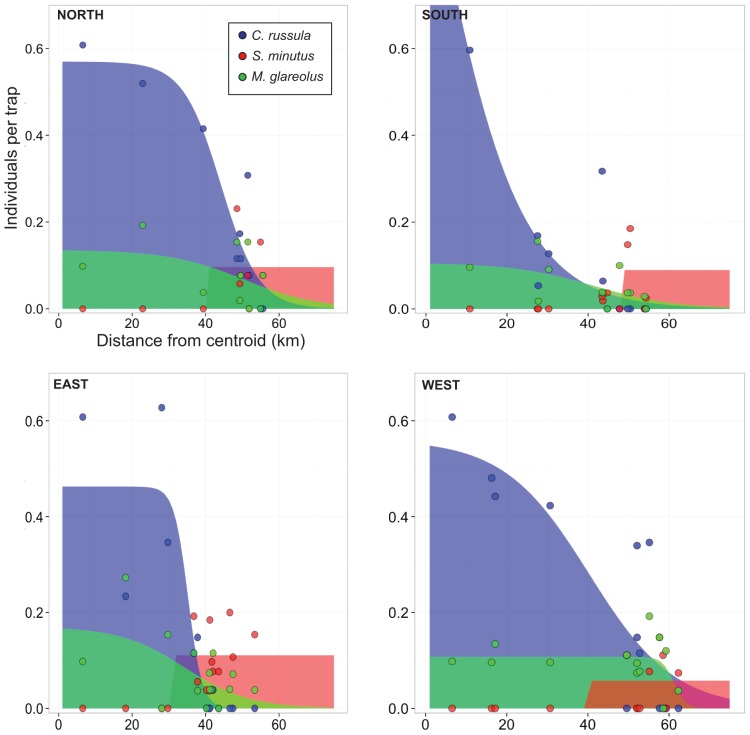
Small mammal species clines in relation to the range of *Crocidura russula*. Species clines for *Crocidura russula* (blue), *Sorex minutus* (red) and *Myodes glareolus* (green) using the 2012 trapping data along the four transects from the centroid (Fig. 1B). The trapping data for the three species are shown as coloured points. Data are from 10, 13, 15 and 12 sampling locations for the North, South, East and West transects, respectively.

*C. russula* was detected at 231 localities (181 via captures/sightings/dead specimens and 50 through recorded presence in bird of prey pellets/nest inspections at individual sites) between 2007 and 2013 (only a single record from 2007 and the species was not recorded in 2006; Fig. 1A). The area of the main distribution range was estimated to be approximately 7,600 km^2^ as of November 2013, an increase of over 300% from the estimate in 2010/2011 [24]. The edge of this range (Zone 2) was identified by a lower abundance of *C. russula* and by the abrupt presence of *S. minutus* (Fig. 2). The two shrew species occurred together in 25 sites within Zone 2 (*S. minutus* was completely absent within Zone 1). Four of these sites were revisited in 2013 and *S. minutus* was only found in two of the four sites. *M. glareolus* abundance was higher in the presence of *C. russula* in three of the four transects examined (Fig. 2; Table S13). No differences were observed in *A. sylvaticus* abundance in relation to the range of *C. russula* (data not shown). Furthermore, *C. russula* records were found outside Zones 1 and 2, with two of these (‘O1’ and ‘O2’; Fig. 1A) being confirmed as established, geographically isolated populations. The species was observed on a single occasion in ‘O3’ in 2010 (S. Perkins, pers. comm.) but was not recorded in the area again in subsequent years despite substantial trapping efforts (Fig. 1A).

**Figure 3:**
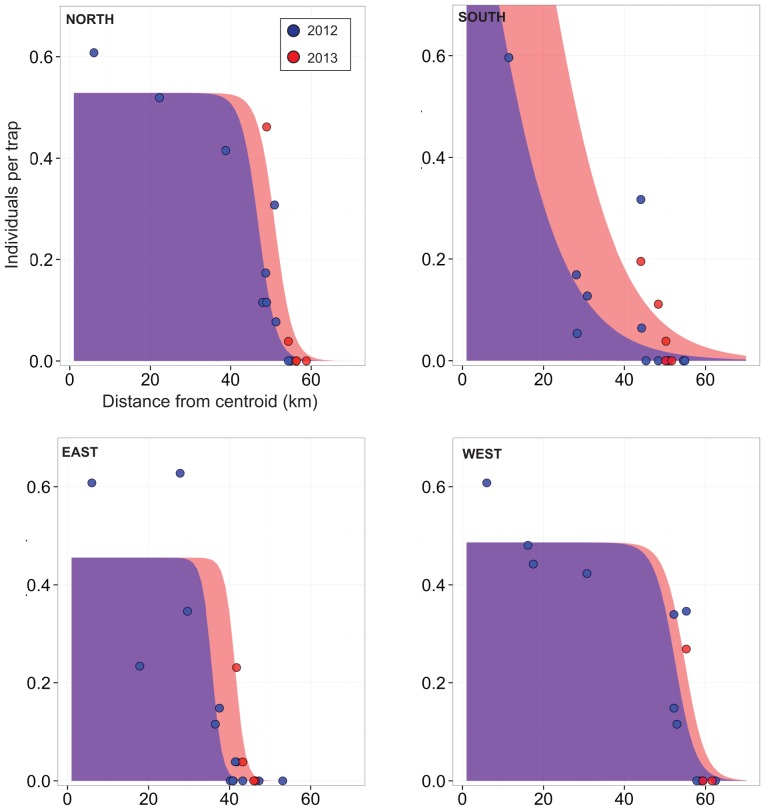
Expansion of the range of *Crocidura russula* 2012–2013. *Crocidura russula* clines for 2012 (blue) and 2013 (red) trapping data (Fig. 1A) along the four transects from the centroid (Fig. 1B). The trapping data for the two years are shown as points. For the north, south, east and west transects There are 15, 18, 19 and 15 data points, and R^2^ values between predicted and observed abundances are 0.85, 0.73, 0.83 and 0.72, respectively.

Using all the available data collected from 2008–2013 (Fig. 1A) gave an estimate for the radial rate of expansion at 5.5 km/yr (±0.8 km/yr) and an expansion estimated to date from 2004 (Fig. S3). Using only the summer trapping data from 2012 and 2013, an increase in range was observed in three out of the four transects (no increase was observed in the West transect; Fig. 3, Table 1). The increase was greatest in the South transect (Fig. 3, Table 1). The estimated velocity of the range expansion and times since expansion differed across the four transects (Table 1). The North, East and West transects had point estimates of range expansions from Method 1 of 2.4–5.7 km/yr (Table 1). For Method 1 the point estimate of the range expansion for the South transect was 14 km/yr, but with a large uncertainty (S.E. = 7 km/yr). This obviously led to different estimates for the time since the range began to expand, ranging from 4 years (inter-quartile range of 2–7 years) for the South transect, to 17 years (inter-quartile range of 11–31 years) for the West transect from 2012 (Table 1). Considering the upper and lower quartiles for time since expansion, the expansion could have begun between 1981 and 2010 (depending on the transect; Table 1). Method 2 gave point estimates for range expansion rates in the range 0.5–7.1 km/yr (Table 1; Figs. S4–S7). The one standard error intervals of all these estimates overlap with those from Method 1. Method 3 gave single point estimates of range expansion between 2012 and 2013 in the range 0–5.6 km/yr (Table 1).

### Species and Habitat Associations

A total of 653 *C. russula*, 258 *S. minutus*, 381 *M. glareolus* and 142 *A. sylvaticus* individuals were recorded during the 2012 summer survey. *C. russula* was recorded at 63 sites out of 123 (51%), *S. minutus* at 81 (66%), *M. glareolus* at 99 (80%) and *A. sylvaticus* at 64 (52%). Correlations between captures of *S. minutus* and the other species caught within a site showed no evidence that our trapping methodology was biasing our abundance estimates. In sites where both *S. minutus* and *C. russula* were caught, there was no significant negative correlation between the trapping probability of *C. russula* and *S. minutus* (r = −0.2, df = 24, *p* = 0.3). There was also no significant correlation between the trapping probability of *S. minutus* and any other small mammal (r = −0.003, df = 24, *p* = 0.99).

**Figure 4:**
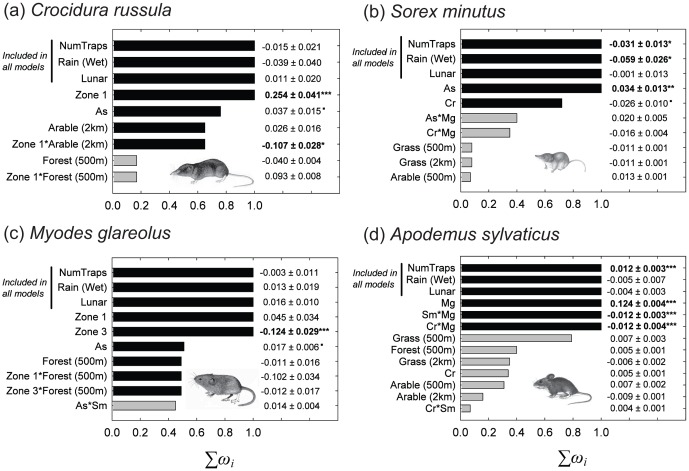
The model averaged terms (from selected models with ΔAICc≤2) for models of the abundance of (a) *Crocidura russula* (Cr) (b) *Sorex minutus* (Sm) (c) *Myodes glareolus* (Mg) and (d) *Apodemus sylvaticus* (As). Bars show the relative importance of each term (ranked in order of the sum of their Akaike weights, Σ*ω_i_*). Black bars indicate terms in the best-approximating model (ΔAICc = 0). NumTraps, Rain and Lunar were included in all models to control for confounding influences. Standardized regression coefficients (*β*) ±1 standard error (SE) are shown to the right of each bar. Significance levels from the best-approximating model are indicated as ^•^ *p*<0.1, * *p*<0.05, ** *p*<0.01 and *** *p*<0.001, and significant terms at *p*<0.05 are shown in bold. The R^2^ values of observed versus fitted values for each best approximating model are 0.53, 0.22, 0.28 and 0.90 for *C. russula*, *S. minutus*, *M. glareolus* and *A. sylvaticus*, respectively.

Model averaging the spatial models showed that *C. russula* abundance was associated positively with *A. sylvaticus* abundance but showed no association with the other species (Fig. 4; Table S5). *C. russula* was negatively associated with arable land at the 2 km scale in Zone 1 but this association was lost in Zone 2. The species was positively associated with forest at the 500 m scale in Zone 1 but the species was negatively associated with the same habitat at the same scale in Zone 2. The abundance of *S. minutus* was negatively associated with *C. russula* and positively associated with *A. sylvaticus* (Fig. 4; Table S6). The interaction of *M. glareolus* and *A. sylvaticus* had a weak positive association with *S. minutus* and the interaction of *C. russula* and *M. glareolus* had a weak negative association. A weak negative association with natural grassland was also found. *M. glareolus* abundance was greater in Zone 1 than either Zone 2 and 3 (Fig. 4; Table S7). *M. glareolus* was positively associated with *A. sylvaticus* and negatively associated with forest in Zone 1 at the 500 m scale but this association with forest was lost within Zones 2 and 3. *A. sylvaticus* showed no relationships with the Zones and had the strongest associations with the other species (and their interactions; Fig. 4; Table S8). It was positively associated with *M. glareolus* but this positive association is reduced in the presence of either *C. russula* or *S. minutus* (negative interactions between shrew species and *M. glareolus*).

## Discussion

*C. russula* is undergoing a rapid range expansion in Ireland. Utilizing data from multiple sources spanning between 2006 and 2013 (Fig. S2) has revealed that the species occupies a large area, considering that it was only being discovered in bird of prey pellets collected in 2007 (Fig. 1A). Given the size of its current range (∼7,600 km^2^), the species was likely present in Ireland prior to 2007 [18], [24], [41]. It is unclear when exactly the species was introduced to Ireland as small mammal trapping in the vicinity of the current range failed to detect the species in the late 1990s and early 2000s [21], [28] but analyses of pine marten (*Martes martes*) diet from samples taken on unspecified dates between 2005 and 2007 did demonstrate that the species was present in several localities [41].

Estimating the rate of expansion from all data gives a value of 5.5±0.8 km/yr, and points to the expansion beginning around 2004 (Fig. S3). Because this rate of expansion is at the upper end of expectations for the species and was based on different detection methods (direct trapping, sightings and bird of prey diet; Fig. S2) and at different times of year, we independently tested this further in 2013 at four areas where the edge of the range was well established in 2012 (Fig. 1A). This edge is indicated by a lower abundance (the number of individuals caught per trap) of *C. russula* and the occurrence of *S. minutus* (Fig. 2). Method 1 gave velocity point estimates of 2.4–14 km/yr, while Methods 2 and 3 gave estimates of 0.5–7.1 km/yr and 0–5.6 km/yr, respectively (Table 1). The variability we documented in velocity between transects is likely due to the effects of landscape features on *C. russula* dispersal ability [30]. The estimate from the West transect was lower due to the fact that the species had not expanded between 2012 and 2013 (Table 1; Fig. 3), likely due to the presence of a significant river barrier in the landscape (without a nearby bridge). Similarly, the largest estimate of 14±7 km/yr (5.6 km/yr using Method 2) was in the South transect and was also likely due to a barrier effect, but in a different way. *C. russula* was present in large numbers at the edge of its range along the South transect in 2012 (having the highest abundance at an edge site of the four transects; Figs. 2 and 3), having encountered a significant barrier in the form of a bisected mountainous landscape, a town, a national road, and the Blackwater River in particular. By 2013, the species had crossed this river (a bridge was present in this instance) and had spread further than the other three transects. This build up of individuals has likely led to more individuals dispersing in search of new territories, leading to a higher rate of successful colonization and establishment. The estimate of 14±7 km/yr is likely a reflection of assuming a fixed shape of the cline between 2012 and 2013, rather than a true reflection of the upper limit of the expansion rate for *C. russula*. The point estimate from Methods 2 and 3 of 7.1 and 5.6 km/yr may be more indicative of the expansion rate in this case (although Method 3 is susceptible to bias from outliers because it is based on the maximum from a sample). Nevertheless, the expansion rate across all years (5.5±0.8 km/yr), and the expansion rates in 2012 for the North, West and East transects (2.4–5.7 km/yr for Method 1, 0.5–4.3 km/yr for Method 2 and 0–3.1 km/yr for Method 3) provide independent verification that *C. russula* is expanding its invasive range at a rapid rate.

These rates of expansion are generally faster than that of *M. glareolus* in Ireland, which was estimated to be spreading at a rate of 2.23–2.63 km/yr after being introduced in the early 1900s [30]. *C. russula* is considered to be a species with limited dispersal from natal sites [46], [47] and indirect estimates of dispersal distances for *C. russula* based on habitat-quality modelling have suggested average distances of 800 m per generation [48]. Direct estimates of dispersal distances during a single breeding session within the breeding season (over a period of 2–3 months) via mark-recapture and parentage assignments were 350 m and 170 m for females and males, respectively (dispersal is sex-biased towards females in the species at the breeding group level [47]), but were as high as 1.3 km for males [42]. However, dispersal in a saturated setting in the centre of a species distribution would not be equivalent to dispersal into unoccupied habitat at the edge of an expanding range. Long-distance dispersal events could become more important in establishing new populations for the latter [49], [50]. Thus, individuals may be expected to invest more in traits associated with dispersal at the range front [9], [51], [52] and rapid evolution of dispersal traits may lead to a manifold increase in distance/range spread [10]. For example, high estimates of rates of expansions have been observed in the invasive cane toad (*Bufo marinus*) in Australia [51]. Earlier estimates of this expansion (1940s–1960s) were approximately 10 km/yr but this was estimated to be 55 km/yr in recent years [51]. This has been corroborated by radio-tracking studies revealing movements up to 21.8 km in a 30-day period [53]. Cane toads at the invasion front have longer hind limbs than those in longer established populations nearer the point of introduction [51] and the rapid expansion has been attributed to the increase in dispersal ability [52]. This has been demonstrated in small mammals colonizing islands previously, but over longer time scales (∼1,000 years; [54]).

Changing life-history traits such as reproductive strategies may also be important at a range front [9], [10]. *C. russula* can produce up to four litters in the wild from March–September [55], with litter sizes from 2–11 [20]. It is only the first of these litters that generally disperses away from the natal site in the first year [46]. They can reach sexual maturity at 58–71 days (in captivity [20]) and it is only those born early in the breeding season that are considered capable of reproducing in their first year [51]. As is the case for dispersal related traits [51], [52], reproductive strategies may be altered in newly colonised, empty habitats where intraspecific competition is less intensive [31], [56]. It is possible that individuals could be reaching sexual maturity earlier, and subsequent litters of *C. russula* (other than the first only) could be dispersing away from natal sites and reproducing at the range edge, leading to increased population growth and subsequent expansion [9], [56]. The *C. russula* invasion warrants further investigation to determine if increased dispersal ability and shifting reproductive strategies are occurring over these fine temporal and spatial scales and are leading to the rapid expansion of the species in Ireland.

Human-assisted dispersal may also be involved. *C. russula* is appearing well outside its main invasive range in Ireland (Zones 1 and 2), with at least two established, discrete outlier populations (‘O1’ and ‘O2’; Fig. 1A). It is possible that these are independent introductions into Ireland but, given their restricted ranges and their more recent discoveries (Fig. 1A), it is much more likely that these are ‘jumps’ occurring within Ireland. As in continental Europe, the species reaches very high densities in the summer [20] and can occupy human dwellings and farm buildings in large numbers [15] within its invasive range in Ireland (AD McDevitt, pers comm.). This may give the false impression that the species is rapidly expanding its range entirely through its own dispersal when it may in fact be assisted, and may explain why it is expanding faster than *M. glareolus*, a species not associated with humans. The possible mechanism of human-mediated dispersal in *C. russula* is uncertain at present but it could be associated with the transport of livestock and/or horticultural produce.

This rapid range expansion of *C. russula* is a serious concern for its potential impacts on the small mammal community in Ireland. In general, habitat associations at a broad scale were not as important as species interactions in determining the abundances of the small mammal species in and around the range of the invasive shrew (Fig. 4). Indeed, the habitat results provide somewhat contradictory messages. *C. russula* showed a negative association with arable land in Zone 1 but this association decreased moving into Zone 2 where it occurs together with *S. minutus* (Fig. 4). This difference can perhaps be explained by colonizing individuals being less selective as they move into new areas, whereas this habitat is avoided where *C. russula* is established. *C. russula* also displayed a positive association with forests in Zone 1 but this was negative within Zone 2 (these associations were weak however). Montgomery et al. [24] reported a strong negative association with forests in Ireland but the species is known to be common in this habitat in parts of continental Europe [20]. For *S. minutus*, species interactions dominated any additional effect of habitat (Fig. 4). Again, Montgomery et al. [24] showed a strong negative association with coniferous forest and arable land for *S. minutus*, although other data show that the species is generally common in forests (both deciduous and coniferous) in Ireland [27], [34]. *M. glareolus* showed a negative association with forests within Zone 1 but this was lost in Zones 2 and 3 (Fig. 4). Certainly *M. glareolus* is common in forests in its natural range [57]. Finally, our study found that *A. sylvaticus* showed a positive association with natural grassland in the best-approximating model and a weak positive association with forests (Fig. 4). Given the dominance of pasture on the Irish landscape (see Methods; [24]), it is perhaps not surprising that it is difficult to distinguish between the remaining habitat types as determinants of small mammal species abundances. Forests cover ∼10% of the landscape in Ireland [58] and peatland is more prevalent in the west (outside the current range of *C. russula*). *S. minutus* are certainly abundant on peatland in Ireland [27], [59] but peatland was not included here because it was a rare habitat within the studied area. Nevertheless, future research could directly investigate the possible importance of these different habitats and if they might act as refugia for *S. minutus* (and *A. sylvaticus*) in Ireland.

The association between invasive and pre-existing species abundances can be particularly revealing in assessing the impacts of the former [24], [60]. *C. russula* abundance was positively associated with that of *A. sylvaticus*, as was *S. minutus* (Fig. 4). This could be due to shrews being generally reliant on the pre-existing underground runs and burrows of rodents [20], [61]. *S. minutus* abundance was negatively associated with *C. russula* where they were present together and *S. minutus* was completely absent at all sites except for those on the edge of the invasive range (Fig. 2). The abrupt appearance of *S. minutus* at the edge of the range of *C. russula* range suggests that the replacement of the species is rapid. Indeed, *S. minutus* was already absent at two of the four edge sites from 2012 that were revisited in 2013. It is important to note that while trapping effort was accounted for in the analysis (NumTraps; Fig. 4), the detection probability in occupancy rates was not because the vast majority of sites were visited only once [62]. It is therefore possible that *S. minutus* was not detected when present. However, Montgomery et al. [24] also noted the absence of *S. minutus* at sites where *C. russula* was present in 2010/2011. In addition, Montgomery et al. [24] provided evidence that the presence of both invasive species together (*C. russula* and *M. glareolus*) was having cumulative negative impacts on *S. minutus* and *A. sylvaticus* in Ireland (‘invasional meltdown’). Here, the interaction between *C. russula* and *M. glareolus* had a weak negative association with *S. minutus* abundance in comparison to the negative association of *C. russula* alone (Fig. 4). Similarly, *A. sylvaticus* abundance was negatively associated by the interaction of the two invasive species (Fig. 4). *M. glareolus* abundance increased significantly in the presence of *C. russula* in three out of the four transects examined (Fig. 2; Table S13). These results are consistent with the possibility of an invasional meltdown [24]. At present however, it is important to note that we do not have sufficient data from sites where *C. russula* is present and *M. glareolus* is absent (only three sites out of 123) and neither have we sampled outside of the range where the two invasive species are present (even though we had 22 sites where both *C. russula* and *M. glarelous* were absent and *S. minutus* was present) to conclusively ascertain if the cumulative effect of the two invasive species is negatively impacting *S. minutus* [24]. It is also unclear exactly how the cumulative effects of the two invasive species would impact upon *S. minutus* and *A. sylvaticus*. There is certainly dietary overlap between the insectivorous *C. russula* and *S. minutus* [20], [61], and the omnivorous *M. glareolus* and *A. sylvaticus* [63]. Arthropods form a more substantial part of both the diets of *M. glareolus* and *A. sylvaticus* during the summer months in particular [24] so this could lead to direct competition for prey. Therefore, an investigation into diet overlap between the four species is warranted. The arrival of these invasive species could also lead to an increase in predator density, leading to the further decline of the less abundant prey through incidental capture (‘apparent competition’ [64]). As with diet overlap between species, further work is necessary on the abundance of predators and the composition of these small mammal species in their diet in this region.

What we know for certain based on this study, is that the presence of *C. russula* is associated with the decline and apparent extirpation of *S. minutus* (Figs. 2 and 4). *C. russula* is known to have had negative impacts on another shrew species, *Crocidura leucodon*, displacing it in Switzerland in association with the range expansion of *C. russula* during the 20^th^ century [65]. However, *C. russula* did not competitively exclude another similarly sized shrew (*Sorex coronatus*) in the same region [15]. Regional coexistence of *S. coronatus* and *C. russula* was maintained by a degree of habitat specialization, with local coexistence facilitated by dispersal from these source habitats [15]. It is unclear what impacts *C. russula* has on *S. minutus* in continental Europe, as *S. minutus* is generally uncommon [25]–[27]. Lower densities of *S. minutus* in some parts of its range have been attributed to interspecific competition with *S. araneus* (sibling species to *S. coronatus*) [66] and the larger body size of *S. araneus* has been proposed as a major factor in competitive interactions between *S. minutus* and *S. araneus* [12], [13]. However, other studies have found no evidence of competition between *S. minutus* and *S. araneus* [14], [67]. *S. minutus* and *C. russula* are both common in the same hedgerows along agricultural land on the island of Belle Île (Fig. S1). *S. coronatus* is found on the mainland but is notably absent from this island, and both *M. glareolus* and *A. sylvaticus* are present in this small mammal community [19], making it similar to the current situation found in Ireland. Therefore, coexistence between *S. minutus* and *C. russula* is clearly possible on an island with habitats similar to those present in Ireland.

The following scenario can be developed: *S. minutus* may be specializing on smaller prey items under competition from the larger *C. russula* and *S. araneus/coronatus* species where they occur together in mainland Europe [13], [68]. Thus, there may have been partial competitive release on Belle Île due to the absence of a large *Sorex* species, allowing an increased abundance of *S. minutus* on the island, despite the presence of the large *Crocidura* species (Fig. S1). When *S. minutus* colonised Ireland the competitive release would potentially have been stronger than on Belle Île due to the absence of both large *Sorex* and *Crocidura* species. *S. minutus* in Ireland take a wider variety of prey items than *S. minutus* in Britain [34] and was also observed taking larger prey species when *S. araneus* was removed in controlled experiments [69]. It is therefore possible that *S. minutus* in Ireland have been able to exploit larger prey items than elsewhere in its range, due to the absence of both large *Sorex* and *Crocidura* species. If this has evolved into a dependence on larger prey items on the island [70], this may at least partly explain the impact of the introduction of *C. russula* (which eats a wide range of invertebrates [20]) to Ireland. *C. russula*, through its competitive superiority in eating large prey items, could have negative impacts on *S. minutus* in Ireland (as an example of exploitative competition [4]). It would therefore be beneficial to investigate the particular diet of each species where they co-exist and in isolation, with the additional use of removal experiments [69] to ascertain the potential negative impacts of *C. russula* on *S. minutus*.

The sheer speed of the invasion of *C. russula* in Ireland is likely to be an important feature. *S. minutus* has been Ireland's sole shrew species for thousands of years [21], [22]. The rapid rate at which *C. russula* is expanding and its much higher densities than *S. minutus* [16], [20] means that *S. minutus* has little time to adapt to its new competitor. The situation mirrors that of the cane toad invasion in Australia where some species are similarly heavily impacted by the invader because of the speed of the invasion and large numbers of the invader [71]. Increased habitat specialisation of *S. minutus* as a response to the new invader [12], [15] may not be possible because of the relatively homogenous nature of the Irish landscape. Habitat types that may act as refugia (such as peatland and woodland) are of a fragmentary nature in Ireland. There may not be sufficient landscape complexity to allow niche partitioning between the two shrew species and a viable metapopulation structure in *S. minutus* in the presence of *C. russula*. However, the full response of *S. minutus* in different habitats in Ireland is not clear. For instance, the *C. russula* invasion has not yet reached the primary areas of peatland in Ireland.

Future research should focus further on the direct interactions and resource utilization between *C. russula* and *S. minutus* in Ireland. More studies would also be desirable on the potential cumulative impacts of the two invasives, *C. russula* and *M. glareolus* [24], exploiting the fact that *C. russula* has recently become established outside of the range of *M. glareolus* (outlier population ‘O1’; Fig. 1A). Unfortunately, the displacement of *S. minutus* may continue in Ireland as *C. russula* carries on spreading rapidly, with the invader only being temporarily hindered by rivers and other barriers (Fig. 3; [30]). Based simply on the size of the island (∼85,000 km^2^), and using the expansion rate from the linear regression approach (5.5 km/yr), *C. russula* will have colonized the whole island before 2050. Given that eradication is unfeasible at this point because of the large area that *C. russula* occupies, this may mean that Ireland's small offshore islands (of which *S. minutus* inhabits many [61]) will become an important long-term refuge for Irish *S. minutus*.

## Supporting Information

Figure S1 **Mean relative abundance (±SD) of the same four small mammal species in Belle Île, France.** *Cr*: *Crocidura russula*; *Sm*: *Sorex minutus*; *Mg*: *Myodes glareolus*; *As*: *Apodemus sylvaticus* trapped at four sites in Belle Île in October 2006 [27].
(DOCX)

Figure S2 **All records relating to the distribution of** ***Crocidura russula*** **subdivided by type from 2006–2013.** ‘Sighting (living/dead)’ represents an observation of a living or dead *C. russula*. ‘Trapping’ and ‘Bird of Prey’ represent potential opportunities to detect *C. russula* by trapping or analysis of bird of prey pellets/nest inspections; these generated either positive or negative records for the analyses conducted in this paper.
(DOCX)

Figure S3 **The radial range (km) of the** ***Crocidura russula*** **distribution as a function of time (years).** The range is defined as (*A*/π)^0.5^ where the locations of all *C. russula* presences up to a certain time are used to calculate the area, *A*, of the *C. russula* minimum convex polygon. Linear regression gives a slope of 5.5±0.8 km/yr (the grey shading represents the 95% confidence region).
(DOCX)

Figure S4 **Splines fitted to the distance from centroid and the** ***C. russula*** **abundance along the East transect.** The shaded region represents 1 standard error in estimates of the distance. Data (n = 17, shown as circles) were required to be at least 25 km from the centroid of Zone 1 in order to focus upon the invasion front. The results for 2012 and 2013 are in blue and red respectively. The velocity of the front is estimated to be 3.0±2.1 km/yr.
(DOCX)

Figure S5 **Splines fitted to the distance from centroid and the** ***C. russula*** **abundance along the West transect.** The shaded region represents 1 standard error in estimates of the distance. Data (n = 12, shown as circles) were required to be at least 25 km from the centroid of Zone 1 in order to focus upon the invasion front. The results for 2012 and 2013 are in blue and red respectively. The velocity of the front is estimated to be 0.5±1.7 km/yr.
(DOCX)

Figure S6 **Splines fitted to the distance from centroid and the** ***C. russula*** **abundance along the North transect.** The shaded region represents 1 standard error in estimates of the distance. Data (n = 13, shown as circles) were required to be at least 25 km from the centroid of Zone 1 in order to focus upon the invasion front. The results for 2012 and 2013 are in blue and red respectively. The velocity of the front is estimated to be 4.3±1.6 km/yr.
(DOCX)

Figure S7 **Splines fitted to the distance from centroid and the** ***C. russula*** **abundance along the South transect.** The shaded region represents 1 standard error in estimates of the distance. Data (n = 17, shown as circles) were required to be at least 25 km from the centroid of Zone 1 in order to focus upon the invasion front. The results for 2012 and 2013 are in blue and red respectively. The velocity of the front is estimated to be 7.1±3.3 km/yr.
(DOCX)

Table S1 **Positive and negative records of** ***Crocidura russula*** **used to estimate the range expansion between 2008 and 2013.** The year and co-ordinates of positive (1) and negative (0) records of *C. russula* in Ireland. Data was obtained from the National Biodiversity Database Centre (NBDC) in Ireland, and through live-trapping, and from bird of prey pellet and nest inspection data (this study).
(XLSX)

Table S2 **The abundances of the four small mammal species captured during live-trapping in 2012 and 2013.** The number of individuals caught per trap in each locality. The associated number of traps (NumTraps), lunar phase for 2012 (Lunar) and whether or not it rained during the trapping session for 2012 (Rain) at each locality.
(XLSX)

Table S3 **A description of the variables in the models for species and habitat associations.** Variables NumTraps, Rain and Lunar were included in all models as control variables.
(DOCX)

Table S4 **The maximal models for the abundance of** ***Sorex minutus*** **,** ***Crocidura russula*** **,** ***Myodes glareolus*** **and** ***Apodemus sylvaticus*** **.** All continuous explanatory variables were centred (zero mean) and scaled (unit variance). All valid sub-models were also fitted and model averaging using AICc was performed to find the set of best fitting models and their coefficients.
(DOCX)

Table S5 **Model averaging results for the abundance of** ***Crocidura russula*** **.** Best-approximating model in bold. Averages are over four selected models. Columns show the term in the model, the average coefficient for that term, the averaged standard error (s.e.), the importance of each term over all selected models (i.e. the sum of Akaike weights Σ*ω_i_*) and the p-value of terms in the best-fit model. All variables are scaled to have a mean of zero and unit variance. The R^2^ values of observed versus fitted values for the best approximating model and a null model with only control variables (NumTraps, Rain and Lunar) are 0.53 and 0.01 respectively.
(DOCX)

Table S6 **Model averaging results for the abundance of** ***Sorex minutus*** **.** Best-approximating model in bold. Averages are over 10 selected models. Columns show the term in the model, the average coefficient for that term, the averaged standard error (s.e.), the importance of each term over all selected models (i.e. the sum of Akaike weights Σ*ω_i_*) and the p-value of terms in the best-fit model. All variables are scaled to have a mean of zero and unit variance. The R^2^ values of observed versus fitted values for the best approximating model and a null model with only control variables (NumTraps, Rain and Lunar) are 0.22 and 0.14 respectively.
(DOCX)

Table S7 **Model averaging results for the abundance of** ***Myodes glareolus*** **.** Best-approximating model in bold. Averages are over four selected models. Columns show the term in the model, the average coefficient for that term, the averaged standard error (s.e.), the importance of each term over all selected models (i.e. the sum of Akaike weights Σ*ω_i_*) and the p-value of terms in the best-fit model. All variables are scaled to have a mean of zero and unit variance. The R^2^ values of observed versus fitted values for the best approximating model and a null model with only control variables (NumTraps, Rain and Lunar) are 0.28 and 0.01 respectively.
(DOCX)

Table S8 **Model averaging results for the abundance of** ***Apodemus sylvaticus*** **.** Best-approximating model in bold. Averages are over 21 selected models. Columns show the term in the model, the average coefficient for that term, the averaged standard error (s.e.), the importance of each term over all selected models (i.e. the sum of Akaike weights Σ*ω_i_*) and the p-value of terms in the best-fit model. All variables are scaled to have a mean of zero and unit variance. The R^2^ values of observed versus fitted values for the best approximating model and a null model with only control variables (NumTraps, Rain and Lunar) are 0.90 and 0.004 respectively.
(DOCX)

Table S9 **The four selected models (ΔAIC<2) for the abundance of** ***Crocidura russula*** **used for model averaging in Table S5.** Shown are the AIC_c_, ΔAIC_c_ and Akaike weights, *w_i_* for each model. For all models the response variable is Cr^0.5^.
(DOCX)

Table S10 **The 10 selected models (ΔAIC<2) for the abundance of** ***Sorex minutus*** **used for model averaging in Table S6.** Shown are the AIC_c_, ΔAIC_c_ and Akaike weights, *w_i_* for each model. For all models the response variable is Sm^0.5^.
(DOCX)

Table S11 **The four selected models (ΔAIC<2) for the abundance of** ***Myodes glareolus*** **used for model averaging in Table S7.** Shown are the AIC_c_, ΔAIC_c_ and Akaike weights, *w_i_* for each model. For all models the response variable is Mg^0.5^.
(DOCX)

Table S12 **The 21 selected models (ΔAIC<2) for the abundance of** ***Apodemus sylvaticus*** **used for model averaging in Table S8.** Shown are the AIC_c_, ΔAIC_c_ and Akaike weights, *w_i_* for each model. For all models the response variable is As^0.5^.
(DOCX)

Table S13 **The parameter estimates for clines fitted to the 2012 trapping data along each of four transects for three species of small mammal (** ***Apodemus sylvaticus*** **not shown).** *x_0_* is the asymptotic number of individuals per trap far from the cline, σ is the width of the cline (km) and *d_1/2_* is the distance (km) from the centroid of Zone 1 at which the number of individuals per trap equals *x*_0_/2.
(DOCX)

File S1 **Further details of spatial models for the abundance of each small mammal species, and modelling clines in species abundance using generalized additive models (GAMs).**
(DOCX)
