# Supplementary material for: Invading and Expanding: Range Dynamics and Ecological Consequences of the Greater White-Toothed Shrew (Crocidura russula) Invasion in Ireland
Source: PLoS One. 2014 Jun 23;9(6):e100403. doi: 10.1371/journal.pone.0100403 (PMC4067332; doi:10.1371/journal.pone.0100403)
Supplement: Figure S1 — Mean relative abundance (±SD) of the same four small mammal species in Belle Île, France. Cr: Crocidura russula; Sm: Sorex minutus; Mg: Myodes glareolus; As: Apodemus sylvaticus trapped at four sites in Belle Île in October 2006 [27]. (DOCX) [file pone.0100403.s001.docx]

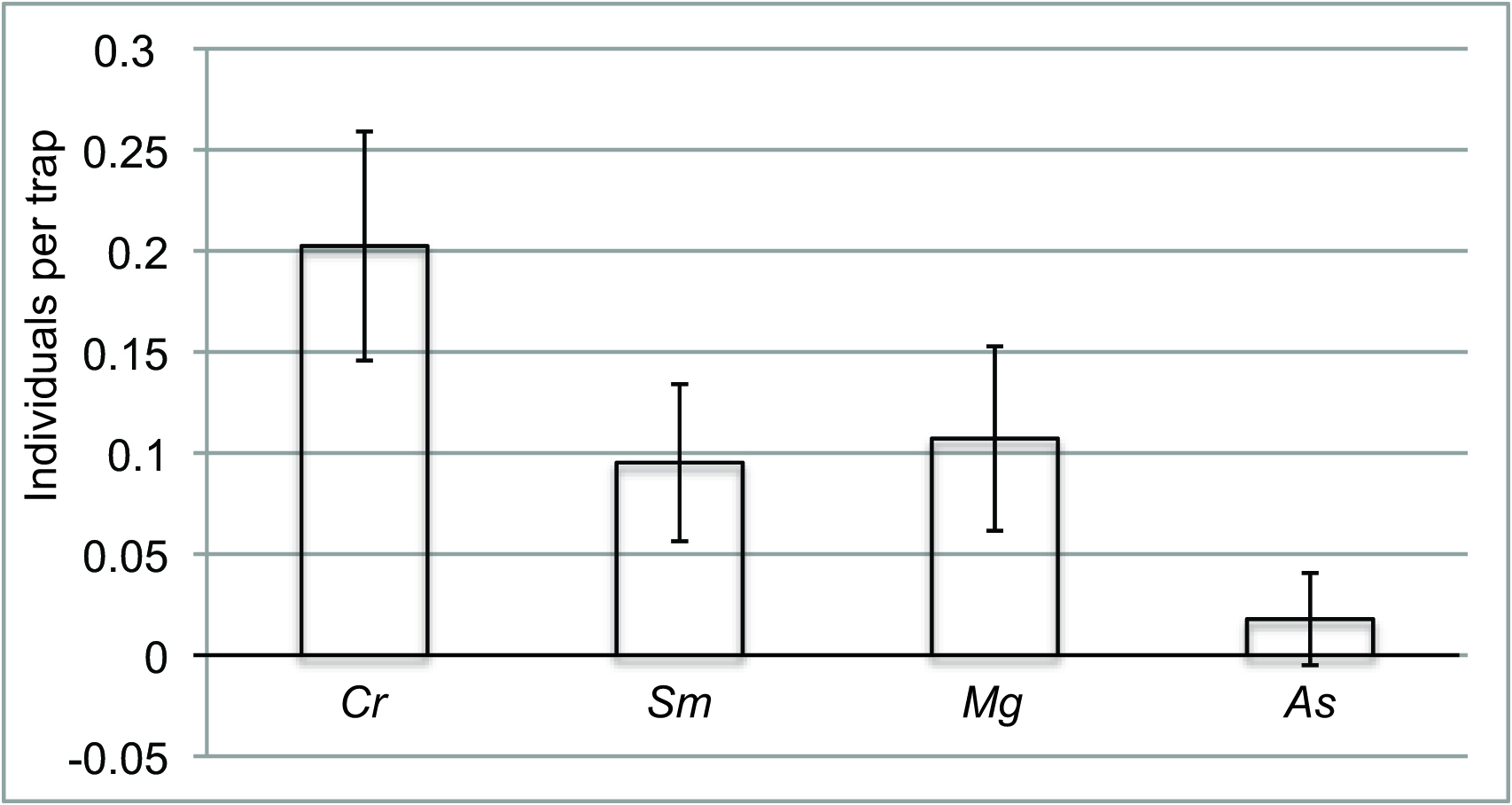


**Figure S1.** Mean relative abundance (±SD) of the same four small mammal species from this study (*Cr*: *Crocidura russula*; *Sm*: *Sorex minutus*; *Mg*: *Myodes glareolus*; *As*: *Apodemus sylvaticus*) trapped at four sites in Belle Île, France in October 2006 [27].
