# Supplementary material for: Invading and Expanding: Range Dynamics and Ecological Consequences of the Greater White-Toothed Shrew (Crocidura russula) Invasion in Ireland
Source: PLoS One. 2014 Jun 23;9(6):e100403. doi: 10.1371/journal.pone.0100403 (PMC4067332; doi:10.1371/journal.pone.0100403)
Supplement: Figure S2 — All records relating to the distribution of Crocidura russula subdivided by type from 2006–2013. ‘Sighting (living/dead)’ represents an observation of a living or dead C. russula. ‘Trapping’ and ‘Bird of Prey’ represent potential opportunities to detect C. russula by trapping or analysis of bird of prey pellets/nest inspections; these generated either positive or negative records for the analyses conducted in this paper. (DOCX) [file pone.0100403.s002.docx]

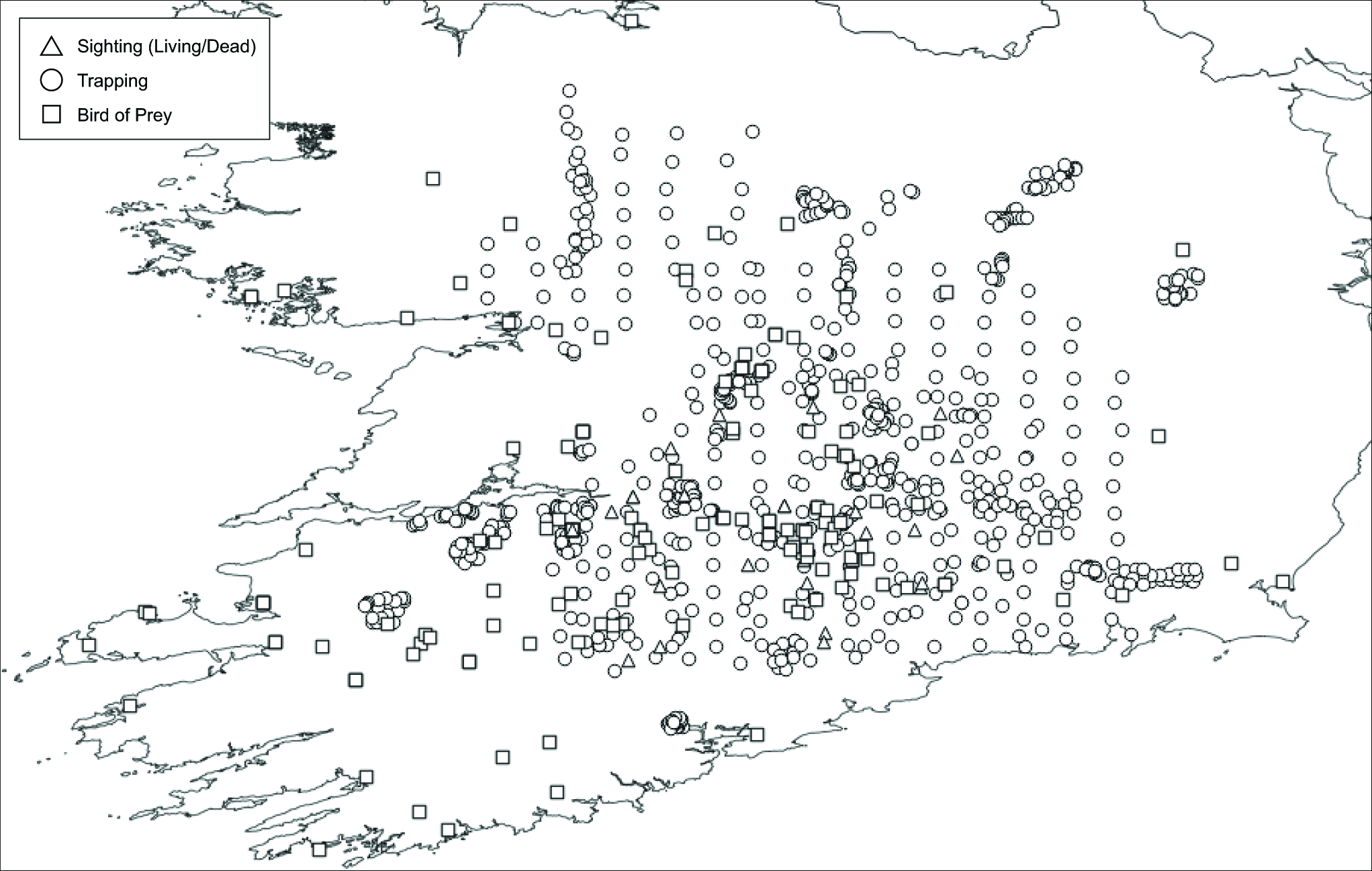


**Figure S2.** All records relating to the distribution of *Crocidura russula* subdivided by type from 2006–2013. ‘Sighting (living/dead)’ represents an observation of a living or dead *C. russula*. ‘Trapping’ and ‘Bird of Prey’ represent potential opportunities to detect *C. russula* by trapping or analysis of bird of prey pellets/nest inspections; these generated either positive or negative records for the analyses conducted in this paper.
