# Supplementary material for: Invading and Expanding: Range Dynamics and Ecological Consequences of the Greater White-Toothed Shrew (Crocidura russula) Invasion in Ireland
Source: PLoS One. 2014 Jun 23;9(6):e100403. doi: 10.1371/journal.pone.0100403 (PMC4067332; doi:10.1371/journal.pone.0100403)
Supplement: Figure S3 — The radial range (km) of the Crocidura russula distribution as a function of time (years). The range is defined as (A/π)0.5 where the locations of all C. russula presences up to a certain time are used to calculate the area, A, of the C. russula minimum convex polygon. Linear regression gives a slope of 5.5±0.8 km/yr (the grey shading represents the 95% confidence region). (DOCX) [file pone.0100403.s003.docx]

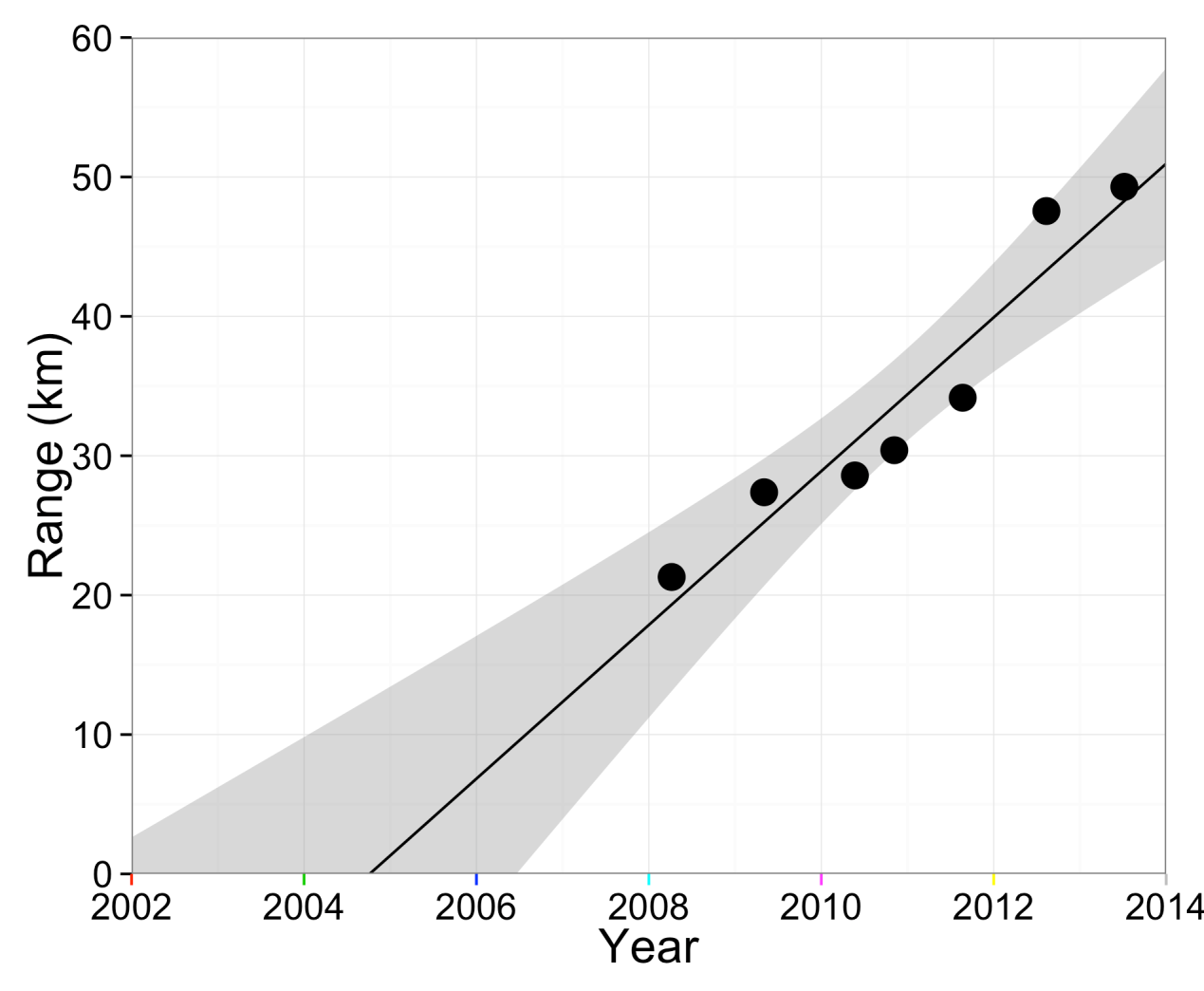


**Figure S3**. The radial range (km) of the *Crocidura russula* distribution as a function of time (years). The range is defined as (*A*/π)^0.5^ where the locations of all *C. russula* presences up to a certain time are used to calculate the area, *A*, of the *C. russula* minimum convex polygon. Linear regression gives a slope of 5.5±0.8 km/yr (the grey shading represents the 95% confidence region).
