# Supplementary material for: Invading and Expanding: Range Dynamics and Ecological Consequences of the Greater White-Toothed Shrew (Crocidura russula) Invasion in Ireland
Source: PLoS One. 2014 Jun 23;9(6):e100403. doi: 10.1371/journal.pone.0100403 (PMC4067332; doi:10.1371/journal.pone.0100403)
Supplement: Figure S4 — Splines fitted to the distance from centroid and the C. russula abundance along the East transect. The shaded region represents 1 standard error in estimates of the distance. Data (n = 17, shown as circles) were required to be at least 25 km from the centroid of Zone 1 in order to focus upon the invasion front. The results for 2012 and 2013 are in blue and red respectively. The velocity of the front is estimated to be 3.0±2.1 km/yr. (DOCX) [file pone.0100403.s004.docx]

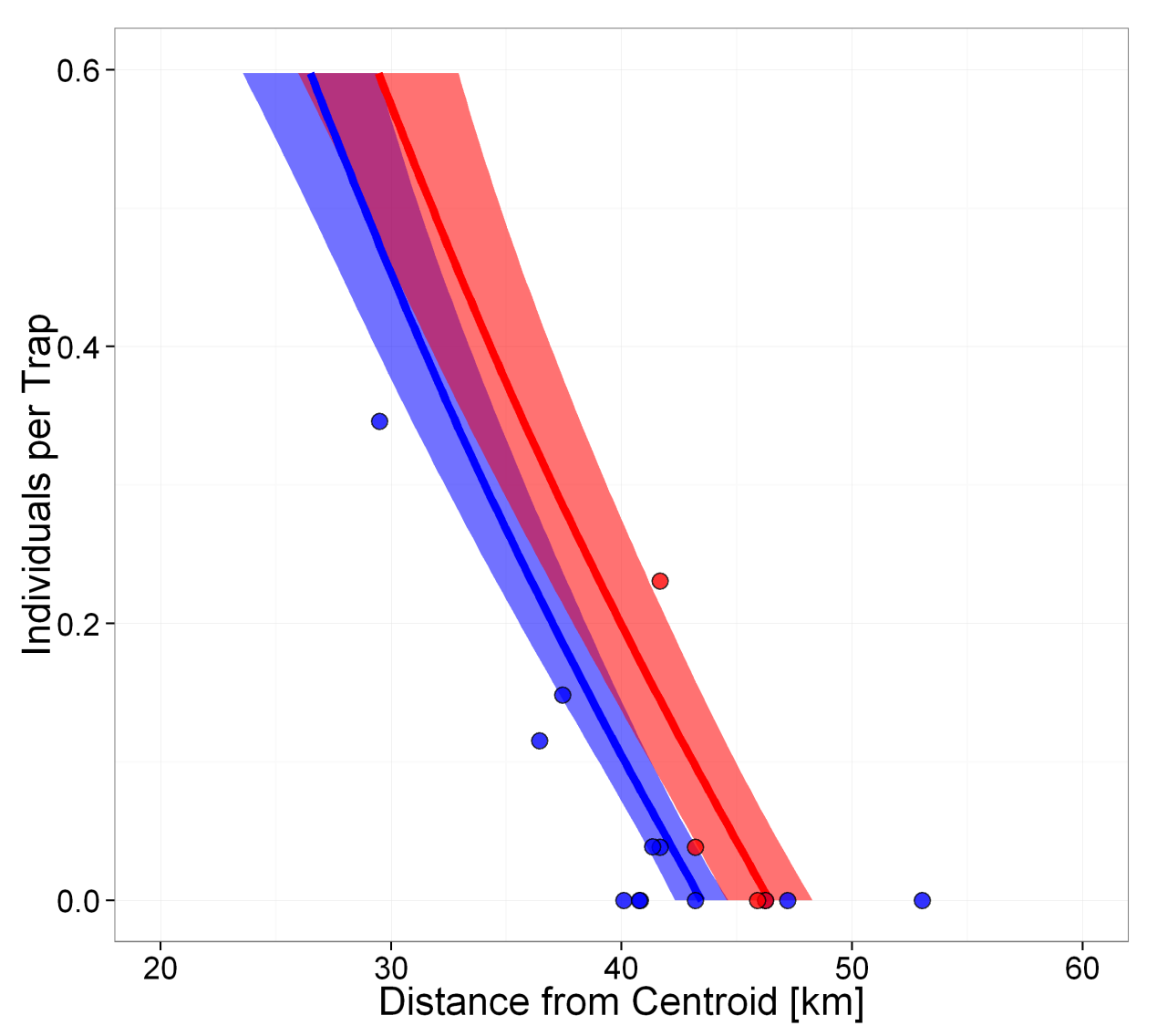


**Figure S4**. Splines fitted to the distance from centroid and the *C. russula* abundance along the East transect (Method 2 in Table 1). The shaded region represents 1 standard error in estimates of the distance. Data (n=17, shown as circles) were required to be at least 25 km from the centroid of Zone 1 in order to focus upon the invasion front. The results for 2012 and 2013 are in blue and red respectively. The velocity of the front is estimated to be 3.0±2.1 km/yr.
