# Supplementary material for: Invading and Expanding: Range Dynamics and Ecological Consequences of the Greater White-Toothed Shrew (Crocidura russula) Invasion in Ireland
Source: PLoS One. 2014 Jun 23;9(6):e100403. doi: 10.1371/journal.pone.0100403 (PMC4067332; doi:10.1371/journal.pone.0100403)
Supplement: Table S3 — A description of the variables in the models for species and habitat associations. Variables NumTraps, Rain and Lunar were included in all models as control variables. (DOCX) [file pone.0100403.s010.docx]

**Table S3.** A description of the variables from the models in Table S4. Variables NumTraps, Rain and Lunar were included in all models as control variables.

| **Variable** | **Description** |
| --- | --- |
| NumTraps | The number of traps used at a sampling site per sampling session |
| Rain | A categorical variable specifying whether or not it rained  WET = rain  DRY = no rain |
| Lunar | The percentage of the moon’s disk that was illuminated |
| Zone | A categorical variable specifying the regions of the *Crocidura russula* invasion (see Fig. 1B in main text)  Zone 1 = *C. russula* caught in this zone but no *Sorex minutus*  Zone 2 = *C. russula* and *S. minutus* caught in this zone  Zone 3 = No *C. russula* caught in this zone |
| arable.500 | The proportion of arable land use within 500 m of the sampling site. Corine codes 211, 242 and 243. (continuous variable) |
| arable.2000 | The proportion of arable land use between 500 m and 2000 m from the sampling site. Corine codes 211, 242 and 243. (continuous variable) |
| forest.500 | The proportion of forest land use within 500 m of the sampling site. Corine codes 311, 312 and 313. (continuous variable) |
| forest.2000 | The proportion of forest land use between 500 m and 2000 m from the sampling site. Corine codes 311, 312 and 313. (continuous variable) |
| grass.500 | The proportion of natural grassland land use within 500 m of the sampling site. Corine codes 321, 322, 324. (continuous variable) |
| grass.2000 | The proportion of natural grassland use between 500 m and 2000 m from the sampling site. Corine codes 321, 322, 324. (continuous variable) |
| Cr | The number of *C. russula* caught per trap per sampling session (continuous variable) |
| Sm | The number of *S. minutus* caught per trap per sampling session (continuous variable) |
| Mg | The number of *Myodes glareolus* caught per trap per sampling session (continuous variable) |
| As | The number of *Apodemus sylvaticus* caught per trap per sampling session (continuous variable) |
