# Supplementary material for: Invading and Expanding: Range Dynamics and Ecological Consequences of the Greater White-Toothed Shrew (Crocidura russula) Invasion in Ireland
Source: PLoS One. 2014 Jun 23;9(6):e100403. doi: 10.1371/journal.pone.0100403 (PMC4067332; doi:10.1371/journal.pone.0100403)
Supplement: Table S4 — The maximal models for the abundance of Sorex minutus , Crocidura russula , Myodes glareolus and Apodemus sylvaticus . All continuous explanatory variables were centred (zero mean) and scaled (unit variance). All valid sub-models were also fitted and model averaging using AICc was performed to find the set of best fitting models and their coefficients. (DOCX) [file pone.0100403.s011.docx]

**Table S4.** The maximal models for the abundance of *Sorex minutus*, *Crocidura russula*, *Myodes glareolus* and *Apodemus sylvaticus*. All continuous explanatory variables (Table S3) were centred (zero mean) and scaled (unit variance). All valid sub-models were also fitted and model averaging using AICc was performed to find the set of best fitting models and their coefficients (see main text and Tables S9-S12).

| **Model** | **Response Variable** | **Explanatory Variables** |
| --- | --- | --- |
| *S. minutus* | Sm^0.5^ | NumTraps + Rain + Lunar +  Zone + arable.500 + forest.500 + grass.500 + arable.2000 + forest.2000 + grass.2000 + Cr + As + Mg +  Zone*(arable.500 + forest.500 + grass.500 + arable.2000 + forest.2000 + grass.2000) + Cr*Mg + Cr*As + Mg*As + Zone*(Cr + As + Mg) |
| *C. russula* | Cr^0.5^ | NumTraps + Rain + Lunar +  Zone + arable.500 + forest.500 + grass.500 + arable.2000 + forest.2000 + grass.2000 + As + Mg +  Zone*(arable.500 + forest.500 + grass.500 + arable.2000 + forest.2000 + grass.2000) + Mg*As + Zone*(As + Mg) |
| *M. glareolus* | Mg^0.5^ | NumTraps + Rain + Lunar +  Zone + arable.500 + forest.500 + grass.500 + arable.2000 + forest.2000 + grass.2000 + As + Cr + Sm  Zone*(arable.500 + forest.500 + grass.500 + arable.2000 + forest.2000 + grass.2000) + Zone*As + As*Cr + Sm*Cr + As*Sm |
| *A. sylvaticus* | As^0.5^ | NumTraps + Rain + Lunar +  Zone + arable.500 + forest.500 + grass.500 + arable.2000 + forest.2000 + grass.2000 + Cr + Sm + Mg +  Zone*(arable.500 + forest.500 + grass.500 + arable.2000 + forest.2000 + grass.2000) + Cr*Sm + Cr*Mg + Mg*Sm |
