# Supplementary material for: Invading and Expanding: Range Dynamics and Ecological Consequences of the Greater White-Toothed Shrew (Crocidura russula) Invasion in Ireland
Source: PLoS One. 2014 Jun 23;9(6):e100403. doi: 10.1371/journal.pone.0100403 (PMC4067332; doi:10.1371/journal.pone.0100403)
Supplement: Table S8 — Model averaging results for the abundance of Apodemus sylvaticus . Best-approximating model in bold. Averages are over 21 selected models. Columns show the term in the model, the average coefficient for that term, the averaged standard error (s.e.), the importance of each term over all selected models (i.e. the sum of Akaike weights Σωi) and the p-value of terms in the best-fit model. All variables are scaled to have a mean of zero and unit variance. The R2 values of observed versus fitted values for the best approximating model and a null model with only control variables (NumTraps, Rain and Lunar) are 0.90 and 0.004 respectively. (DOCX) [file pone.0100403.s015.docx]

**Table S8.** Model averaging results for the abundance of *Apodemus sylvaticus* (best-approximating model in bold). Averages are over 21 selected models. Columns show the term in the model, the average coefficient for that term, the averaged standard error (s.e.), the importance of each term over all selected models (i.e. the sum of Akaike weights Σ*ω_i_*) and the p-value of terms in the best-fit model. All variables are scaled to have a mean of zero and unit variance. The R^2^ values of observed versus fitted values for the best approximating model and a null model with only control variables (NumTraps, Rain and Lunar) are 0.90 and 0.004 respectively.

| Model Term | Coefficient | s.e. | Σ*ω_i_* | p |
| --- | --- | --- | --- | --- |
| **NumTraps** | **0.012** | **0.003** | **1.00** | **0.001** |
| **Rain(Wet)** | **-0.005** | **0.007** | **1.00** | **0.485** |
| **Lunar** | **0.004** | **0.003** | **1.00** | **0.186** |
| **Mg** | **0.124** | **0.004** | **1.00** | **<0.001** |
| **Sm*Mg** | **-0.012** | **0.003** | **1.00** | **<0.001** |
| **Cr*Mg** | **-0.012** | **0.004** | **1.00** | **0.001** |
| grass.500 | 0.007 | 0.003 | 0.79 |  |
| forest.500 | 0.005 | 0.001 | 0.40 |  |
| grass.2000 | -0.006 | 0.002 | 0.35 |  |
| Cr | 0.005 | 0.001 | 0.34 |  |
| arable.500 | 0.007 | 0.002 | 0.31 |  |
| arable.2000 | -0.009 | 0.001 | 0.16 |  |
| Cr*Sm | -0.004 | 0.000 | 0.07 |  |
