# Supplementary material for: Invading and Expanding: Range Dynamics and Ecological Consequences of the Greater White-Toothed Shrew (Crocidura russula) Invasion in Ireland
Source: PLoS One. 2014 Jun 23;9(6):e100403. doi: 10.1371/journal.pone.0100403 (PMC4067332; doi:10.1371/journal.pone.0100403)
Supplement: Table S11 — The four selected models (ΔAIC<2) for the abundance of Myodes glareolus used for model averaging in Table S7. Shown are the AICc, ΔAICc and Akaike weights, wi for each model. For all models the response variable is Mg0.5. (DOCX) [file pone.0100403.s018.docx]

**Table S11.** The four selected models (ΔAIC<2) for the abundance of *Myodes glareolus* used for model averaging in Table S7. Shown are the AIC_c_, ΔAIC_c_ and Akaike weights, *w_i_* for each model. For all models the response variable is Mg^0.5^

| AIC_c_ | ΔAIC_c_ | *w_i_* | Model |
| --- | --- | --- | --- |
| -154.219 | 0.000 | 0.303 | NumTraps + Rain + Lunar + Zone + As + forest.500*Zone |
| -153.976 | 0.243 | 0.268 | NumTraps + Rain + Lunar + Zone + As |
| -153.552 | 0.666 | 0.217 | NumTraps + Rain + Lunar + Zone + forest.500*Zone |
| -153.506 | 0.713 | 0.212 | NumTraps + Rain + Lunar + Zone |
