# Supplementary material for: Invading and Expanding: Range Dynamics and Ecological Consequences of the Greater White-Toothed Shrew (Crocidura russula) Invasion in Ireland
Source: PLoS One. 2014 Jun 23;9(6):e100403. doi: 10.1371/journal.pone.0100403 (PMC4067332; doi:10.1371/journal.pone.0100403)
Supplement: Table S13 — The parameter estimates for clines fitted to the 2012 trapping data along each of four transects for three species of small mammal ( Apodemus sylvaticus not shown). x0 is the asymptotic number of individuals per trap far from the cline, σ is the width of the cline (km) and d1/2 is the distance (km) from the centroid of Zone 1 at which the number of individuals per trap equals x 0/2. (DOCX) [file pone.0100403.s020.docx]

**Table S13.** The parameter estimates for clines (equations 1 and 2 in the main text with *v* set to zero) fitted to 2012 trapping data along each of four transects (see Fig. 2) for three species of small mammal (*Apodemus sylvaticus* not shown). *x_0_* is the asymptotic number of individuals per trap far from the cline, σ is the width of the cline (km) and *d_1/2_* is the distance (km) from the centroid of Zone 1 at which the number of individuals per trap equals *x*_0_/2.

| ***Crocidura russula*** | | | | | |  | ***Sorex minutus*** | | | | |  | ***Myodes glareolus*** | | | | |
| --- | --- | --- | --- | --- | --- | --- | --- | --- | --- | --- | --- | --- | --- | --- | --- | --- | --- |
| **NORTH** | **Estimate** | **SE** | **t value** | **Pr(>\|t\|)** |  |  | **Estimate** | **SE** | **t value** | **Pr(>\|t\|)** |  |  | **Estimate** | **SE** | **t value** | **Pr(>\|t\|)** |  |
| *d_1/2_* | 44 | 3 | 15.60 | <0.001 | *** |  | 41 | >1000 | 0.002 | 1.0 |  |  | 50 | 14 | 3.54 | 0.009 | ** |
| σ | 5 | 2 | 2.54 | 0.04 | * |  | - | - | - | - |  |  | 10 | 23 | 0.45 | 0.7 |  |
| *x_0_* | 0.6 | 0.1 | 8.55 | <0.001 | *** |  | 0.10 | 0.02 | 3.94 | 0.004 | ** |  | 0.14 | 0.07 | 1.87 | 0.1 |  |
| **SOUTH** |  |  |  |  |  |  |  |  |  |  |  |  |  |  |  |  |  |
| *d_1/2_* | 0 | 100 | <0.01 | 1 |  |  | 48 | 43 | 1.13 | 0.3 |  |  | 41 | 17 | 2.48 | 0.04 | * |
| σ | 10 | 10 | 0.98 | 0.4 |  |  | - | - | - | - |  |  | 10 | 12 | 0.81 | 0.4 |  |
| *x_0_* | 2 | 10 | 0.16 | 0.9 |  |  | 0.09 | 0.03 | 3.43 | 0.006 | ** |  | 0.11 | 0.06 | 1.70 | 0.1 |  |
| **EAST** |  |  |  |  |  |  |  |  |  |  |  |  |  |  |  |  |  |
| *d_1/2_* | 35 | 2 | 14.10 | <0.001 | *** |  | 31 | >1000 | 0.002 | 1.0 |  |  | 34 | 12 | 2.83 | 0.02 | * |
| σ | 1.9 | 1.6 | 1.21 | 0.3 |  |  | - | - | - | - |  |  | 8.7 | 8.5 | 1.02 | 0.3 |  |
| *x_0_* | 0.46 | 0.06 | 7.68 | <0.001 | *** |  | 0.11 | 0.02 | 6.79 | <0.001 | *** |  | 0.17 | 0.08 | 2.10 | 0.06 |  |
| **WEST** |  |  |  |  |  |  |  |  |  |  |  |  |  |  |  |  |  |
| *d_1/2_* | 40 | 12 | 3.39 | 0.007 | ** |  | 41 | >1000 | 0 | 1.0 |  |  | 61 | 2 | 26.15 | <0.001 | *** |
| σ | 11 | 7 | 1.54 | 0.2 |  |  | - | - | - | - |  |  | 1.5 | 1.8 | 0.79 | 0.4 |  |
| *x_0_* | 0.56 | 0.15 | 3.64 | 0.005 | ** |  | 0.06 | 0.02 | 3.45 | 0.005 | ** |  | 0.11 | 0.02 | 6.52 | <0.001 | *** |
