# Supplementary material for: Invading and Expanding: Range Dynamics and Ecological Consequences of the Greater White-Toothed Shrew (Crocidura russula) Invasion in Ireland
Source: PLoS One. 2014 Jun 23;9(6):e100403. doi: 10.1371/journal.pone.0100403 (PMC4067332; doi:10.1371/journal.pone.0100403)
Supplement: File S1 — Further details of spatial models for the abundance of each small mammal species, and modelling clines in species abundance using generalized additive models (GAMs). (DOCX) [file pone.0100403.s021.docx]

# Supporting Information

The variables used are described in Table S3. We built spatial models for the number of small mammal species caught per trap at each sampling location (Table S4). All models were fitted by maximum likelihood using generalised least squares (using the nlme package in R) with an exponential spatial error structure. This exponential error structure specifies the correlation between two observations a distance *r* apart as exp(-r/*α*) where *α* is a parameter giving the spatial scale of autocorrelation. The response variable was square-root transformed to compensate for over-dispersion.

Model averaging was done by fitting all valid submodels (e.g. no categorical variable was included in an interaction term with the main effect term also being present) of the maximal models given in Table S4. NumTraps, Rain and Lunar were control variables and always included in a model. The abundance of *S. minutus* was not included as an explanatory variable in the models of *C. russula* because we assume *a priori* that *S. minutus* abundance has no effect upon *C. russula* abundance. The best-approximating model was selecting using AICc and all models having an AICc within two of the best-approximating model were used for model averaging (see main text for details). This gave 4, 10, 4 and 21 selected models for *C. russula*, *S. minutus*, *M. glareolus* and *A. sylvaticus*, respectively. The averaged coefficients for these selected models are shown in Tables S5-S8. For all models the baseline model always corresponds to factor levels of Rain=DRY, Zone=2.

**Modelling clines in species abundance using GAMs**

Our fitted clines assumed a fixed shape of each cline (sigmoidal equation 1 for *C. russula*, *M. glareolus* and *A. sylvaticus* and the step-function equation 2 for *S. minutus*). To test the robustness of our results for expansion rate from the cline analysis we took data from points that were more than 25 km from the centroid (in order to focus upon the invasion front) and fitted generalized additive models (GAMs) using generalized cross validation with the mgcv package in R (Wood SN 2006; Generalized Additive Models: An Introduction with R. Chapman and Hall/CRC). For each transect (North, South, East and West, Fig. 1B) we fitted the model

*d* ~ 1 + Δ*t* + *s*(*x*)

where *x* is the number of individuals per trap, *d* is the distance (km) of a point from the centroid of Zone 1 and Δ*t* is the time since 2012 (yr). The smooth term *s*(*x*) fits a thin plate regression spline that allows the data to determine the shape of the cline. The dimension of the basis used to represent the smooth term was set to *k*=6. The fitted clines for the four transects are shown in Figs. S4-S7. The shape of the cline is assumed to be constant across years and the coefficient for the Δ*t* provides an estimate for the velocity, *v*, of the cline (Table 1).
